# GIMEMA survey on the management of chronic myeloid leukemia patients in the third line and beyond: insights from Italian hematologists

**DOI:** 10.3389/fonc.2025.1697569

**Published:** 2025-11-18

**Authors:** Massimo Breccia, Alfonso Piciocchi, Elisabetta Abruzzese, Daniela Cilloni, Fausto Castagnetti, Monica Messina, Stefano Soddu, Barbara Scappini, Uros Markovic, Mario Annunziata, Alessandra Malato, Gianni Binotto, Olga Mulas, Massimiliano Bonifacio, Paola Fazi, Fabrizio Pane

**Affiliations:** 1 Department of Translational and Precision Medicine, Sapienza University, Rome, Italy; 2 Gruppo Italiano Malattie Ematologiche dell'Adulto (GIMEMA) Foundation, Rome, Italy; 3 Unità Operativa Complessa U.O.C. Ematologia, Ospedale S. Eugenio, Rome, Italy; 4 Ematologia, Azienda Ospedaliera Mauriziano Umberto I, Turin, Italy; 5 Hematology Unit, IRCCS Azienda Ospedaliero, University of Bologna, Bologna, Italy; 6 Department of Medical and Surgical Sciences, University of Bologna, Bologna, Italy; 7 Struttura Ospedaliera Dipartimentale Ematologia, AOU Careggi, Firenze, Italy; 8 Division of Hematology and Bone Marrow Transplantation, Azienda Ospedaliero-Universitaria Policlinico G. Rodolico -San Marco, Catania, Italy; 9 Ospedale Cardarelli, Napoli, Italy; 10 A.O. Ospedali Riuniti Villa Sofia-Cervello-P.O. Cervello, Palermo, Italy; 11 Unit of Hematology and Clinical Immunology, University of Padova, Padova, Italy; 12 Azienda Ospedaliera Brotzu, Presidio Ospedaliero A. Businco, Struttura Complessa Ematologia E CTMO, Cagliari, Italy; 13 Section of Hematology, Department of Medicine, Azienda Ospedaliera Universitaria Integrata di Verona, Verona, Italy; 14 Unità Operativa Complessa Ematologia, AOU Federico II, Napoli, Italy

**Keywords:** chronic myeloid leukemia, later lines, tyrosine kinase inhibitors, prognosis, asciminib

## Abstract

**Background:**

Approximately one-third of chronic myeloid leukemia (CML) patients may develop resistance and/or intolerance to the current therapies and need to switch to later lines of treatment. However, how to choose a later line of therapy is still a matter of discussion.

**Methods:**

A survey was performed by the Gruppo Italiano Malattie Ematologiche dell’Adulto (GIMEMA) to understand how the scenario has changed after the introduction of the first allosteric inhibitor, asciminib, in later lines.

**Results:**

The GIMEMA survey aimed to reassess the Italian approach to third-line or later-line treatments in CML. In the whole cohort of 1,637 patients, to treat resistance, ponatinib was used with a mean of 41% [standard deviation (SD) = 29] and a median of 50% (0–100), while asciminib was used with a mean of 27% (SD = 23) and a median of 25% (0–100). Indeed, to treat intolerance, asciminib was the most used with a mean of 32% (SD = 30) and a median of 30 (0–100), followed by bosutinib with a mean of 25% (SD = 25) and a median of 20 (0–90). Several possible treatment sequences were analyzed, and asciminib emerged as the best third-line treatment.

**Conclusions:**

The survey attempted to understand the major reasons for treatment switch, how tyrosine kinase inhibitors (TKIs) were selected, and which drug was preferred based on patient and disease characteristics. The current algorithm of treatment seems to have changed in both resistant and intolerant CML patients in later lines. The reduction of TKI dose is a current practice to maintain efficacy while reducing the occurrence of side effects.

## Introduction

Chronic myeloid leukemia (CML) is a clonal disorder of hematopoiesis, determined by the acquisition of a proliferative advantage at the level of the pluripotent hematopoietic stem cell. This disease is characterized by the proliferation and progressive accumulation of precursors and mature cells of the granulocyte lineage in the bone marrow and peripheral blood. The reported incidence in industrialized countries is approximately 0.7–1/100,000, with a median age at diagnosis of 57–60 years and a male/female incidence ratio of 1.2–1.7. As regards the prevalence, however, it is estimated that it is approximately 10–12 cases per 100,000 inhabitants, with a constant increase due to the drastic improvement in the survival of these patients. There are no known etiopathogenetic factors; however, exposure to ionizing radiation is certainly one of the most likely factors. Other factors, such as smoking, benzene, pesticide use, and dyes, although lacking specific clinical studies, have been linked to the development of this disease (1, 2). Despite the near-normal life expectancy reached by CML patients with the currently available tyrosine kinase inhibitors approved, some unmet needs are still an open issue in this disease (1, 2). A significant proportion of patients are still forced to change treatment due to resistance or intolerance, and the outcome of this subset of patients remains poor, especially if a treatment switch from second-generation tyrosine kinase inhibitors (TKIs) to a similar-generation drug was performed (2). Recently, asciminib, the first allosteric inhibitor, was approved by the European Medicines Agency (EMA) for use in the third line after the publication of the results of the ASCEMBL trial, a phase 3 study comparing asciminib to bosutinib in later lines (3). Before the final approval, a survey was performed by the Gruppo Italiano Malattie Ematologiche dell’Adulto (GIMEMA) group to analyze how the approach in later lines and the therapeutic algorithm could change. The results clearly showed that, at that time, only 60% of clinicians had experience in the use of the drug, and most treated patients received the drug for previous resistance after a third line. In different scenarios proposed, only approximately 30% of clinicians were ready to use the drug after second-generation TKIs as the second line after imatinib failure in patients without the T315I mutation. Also, approximately 30% were keen to use asciminib as the third-line treatment after the failure of second-generation TKIs and ponatinib as second-line treatments (4). In the meantime, several other studies involving asciminib have been reported: the long-term follow-up of the ASCEMBL study at 156 weeks showed the persistence difference in major molecular response (MMR) (asciminib 33.8% vs. bosutinib 10.5%) with advantage also in terms of MR4 (19.1% vs. 6.6%) and MR4.5 (8.9% vs. 5.3%) (5). The final follow-up of the phase 1 trial, after a median exposure of 5.9 years, reported that 60.9% of patients continued receiving the drug without new safety signals. MMR achieved in 56 out of 115 patients was maintained in 50 patients with a probability of 88% (6). In the third or later lines, the ASC4OPT trial showed a similar advantage of a scheduled dose of 40 mg BID for non-T315I-mutated patients vs. 80 mg QD. At week 48 of follow-up, 42.4% of patients treated with 40 mg BID and 34.5% of patients treated with 80 mg QD reached an MMR (7). Asciminib was tested as a possible frontline treatment in newly diagnosed CML-chronic phase (CP) patients in the randomized ASC4FIRST trial compared to investigator-selected TKIs: 48 weeks of follow-up showed an advantage of asciminib compared to all TKIs in the rate of MMR, the primary endpoint (67.7% vs. 49%), compared to imatinib (69.3% vs. 40.2%), with a reduced rate of discontinuation due to adverse events (5% vs. 13% of all TKIs) (8). After all this, new data were published, and more than 1.5 years after the first survey, we performed another survey to detect how the approaches to third-line treatment had changed after the final placement in the market of the drug.

## Materials and methods

The survey data were collected and managed using the REDCap electronic data capture tools hosted at the GIMEMA Foundation (9). The survey invitation was sent in December 2024, and data were exported in March 2025 as aggregated data with no need for ethical approval or a control group. As in the previously published survey, all centers involved in CML diagnosis and management were invited. Sixty-three centers out of 142 invited distributed across the Italian country compiled the survey: 45 (71%) hospitals and 18 (29%) academic centers (Appendix A). All the centers invited participated in the survey and were considered all centers that previously participated in GIMEMA trials involving CML patients. The median number of years of clinical practice of the participants was 20 (range 2–40). The survey engaged a total number of 8,381 patients, with a median of 100 patients followed up in each involved center and a median of 90 patients followed up per year by participants. Statistical analysis was performed by the GIMEMA data center. The survey required participants to indicate the overall number of patients in third or later lines and the reason for the last switch of treatment. The key objectives of this survey were as follows: 1) evaluate the number of patients treated in the third line in Italy, 2) analyze the main reasons for the treatment switch and the choice of TKIs in different situations, and 3) assess the treatment preferences based on patients’ features. As in the previous survey, participants were asked to respond to different possible algorithms of treatment in resistant and intolerant patients without the T315I mutation, according to different age classes (65–75 and >75 years). In the final survey, participants were also asked about the reduction of dose even in later lines and the possible approach in the future with asciminib as the first-line treatment.

## Results

### Overall population

Of the whole cohort of 8,381 patients referred by all the centers involved in the survey, 1,637 patients (19.5%) were treated in the third line in the last 10 years and 912 (10.9%) in the last 5 years. Of 1,637 patients treated in the third line, 448 (49.1%) switched due to resistance, 331 (36.3%) switched due to true intolerance in the last 5 years, and 133 (14.6%) switched due to initial intolerance and following resistance. Physicians were asked about the main reason for the switch due to intolerance: 30 of them (48%) stated recurrence of side effects, 15 (24%) cardiovascular events, 12 (19%) pulmonary side effects, one (1.6%) metabolic adverse events, and four (6.5%) persistence of low-grade side effects.

### Type of treatment in later lines

**Figure 1:**
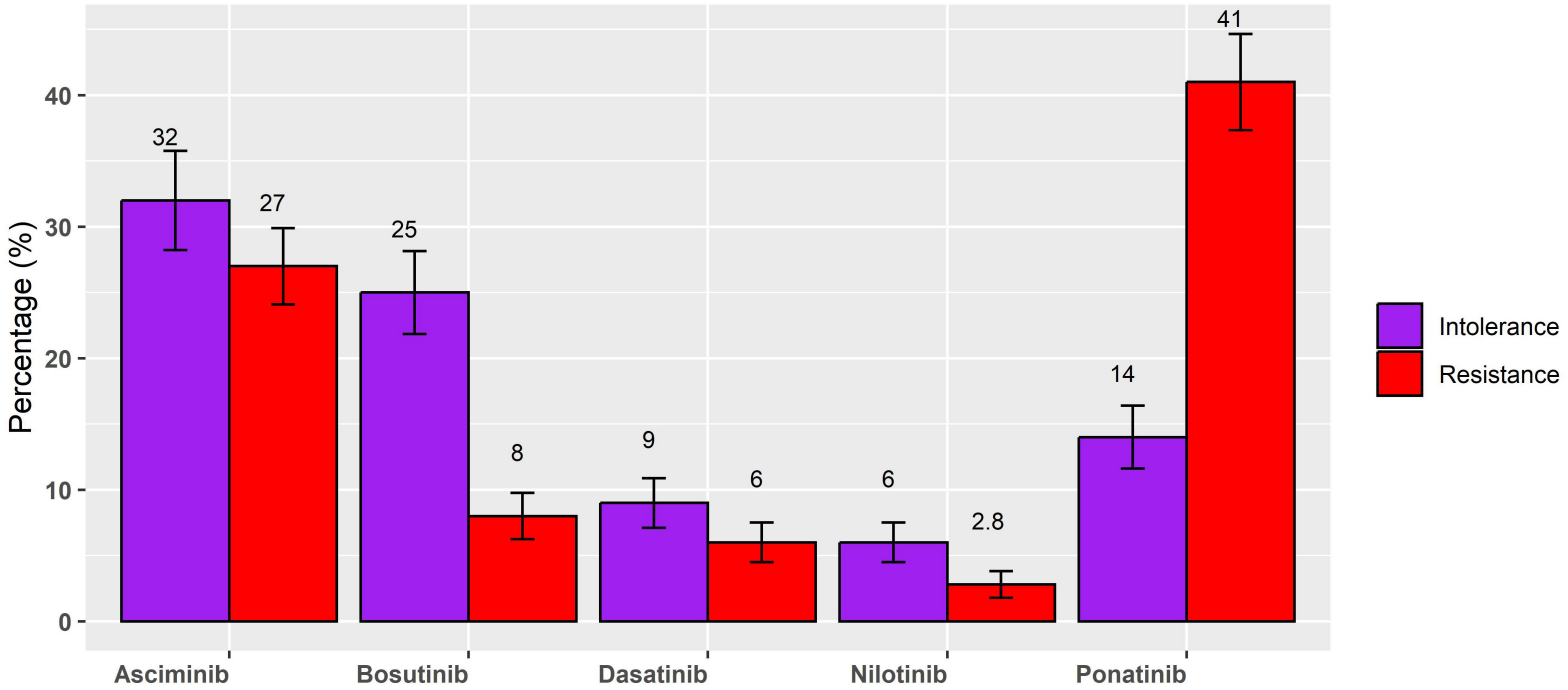
Mean percentage of each drug, stratified for resistance or intolerance for third-line treatment.

Then, participants were asked about the type of drug currently administered. In this respect, the researchers reported the percentage of patients treated with each drug. The mean [standard deviation (SD)] values indicate the average percentage of use, along with the standard deviation, reflecting the variability in usage. The median (range) provides an additional perspective by showing the median percentage of usage and the overall range (minimum to maximum values) observed in the cohort. For resistance, ponatinib was used with a mean of 41% (SD = 29) and a median of 50% (0%–100%), while asciminib was used with a mean of 27% (SD = 23) and a median of 25% (0%–100%). Bosutinib was used with a mean of 8% (SD = 14) and a median of 0% (0%–50%); dasatinib was used with a mean of 6% (SD = 12) and a median of 0% (0%–50%), and nilotinib with a mean of 2.8% (SD = 8) and a median of 0 (0%–50%). For intolerance, asciminib was the most used drug with a mean of 32% (SD = 30) and a median of 30% (0%–100%), followed by bosutinib with a mean of 25% (SD = 25) and a median of 20% (0%–90%). Indeed, ponatinib was used with a mean of 14 (SD = 19) and a median of 6% (range 0%–100%). Dasatinib was used for intolerance with a mean of 9% (SD = 15) and a median of 0% (0%–80%), and nilotinib with a mean of 6% (SD = 12) and a median of 0% (0%–50%). **Figure 1** illustrates the mean percentage usage of each drug, stratified by the reason for third-line treatment: resistance or intolerance. The graph also includes the standard error of the mean (SEM) to provide insight into the variability of drug utilization within each subgroup. This visual representation allows for an easier comparison of drug preference and usage patterns between patients treated for resistance and those treated for intolerance.

### Reduction of the dose as strategy also in later lines

Physicians were asked about the approaches and management of CML in later lines. It emerged that 28 (45%) participants reduced the dose of TKIs (especially bosutinib and ponatinib) even in later lines, but the majority of them (n = 17, 27%) applied the reduction strategy after the achievement of at least MMR (ratio BCR::ABL1 < 0.1%), while 12 (19%) reduced the dose after patients reaching a ratio < 1%. Physicians detailed the reduced dose used for bosutinib in patients with pre-existing cardiovascular comorbidities, and the median dose was 200 mg/day (range 100–300 mg). Indeed, the dose used for asciminib in the CML population with the same cardiovascular conditions did not change as compared to the classic dose suggested for non-T315I-mutated patients in the third line (40 mg BID).

### Possible algorithms of treatment in later lines

Participants were asked about the best sequence to use in a resistant patient without T315I mutation: 26 (41%) physicians used asciminib as the third-line treatment after a previous treatment failure with imatinib as the frontline treatment and a second-generation (2gen) TKI as the second-line treatment, 18 (29%) used asciminib as the third-line treatment after a previous failure of a 2gen TKI frontline treatment and ponatinib as the second-line treatment, and eight (13%) used asciminib as the third-line treatment after the previous failure of two different 2gen TKIs. Only a few clinicians postponed asciminib as the fourth-line treatment: in 6% (n = 4) of the cases if the patient started with imatinib as the frontline treatment and after failure of two previous 2gen TKIs and in 11% (n = 7) of the cases if the patient started with 2gen TKI and failed another 2gen TKI and ponatinib as the third-line treatment.

### Categories of patients who could benefit from asciminib

**Figure 2:**
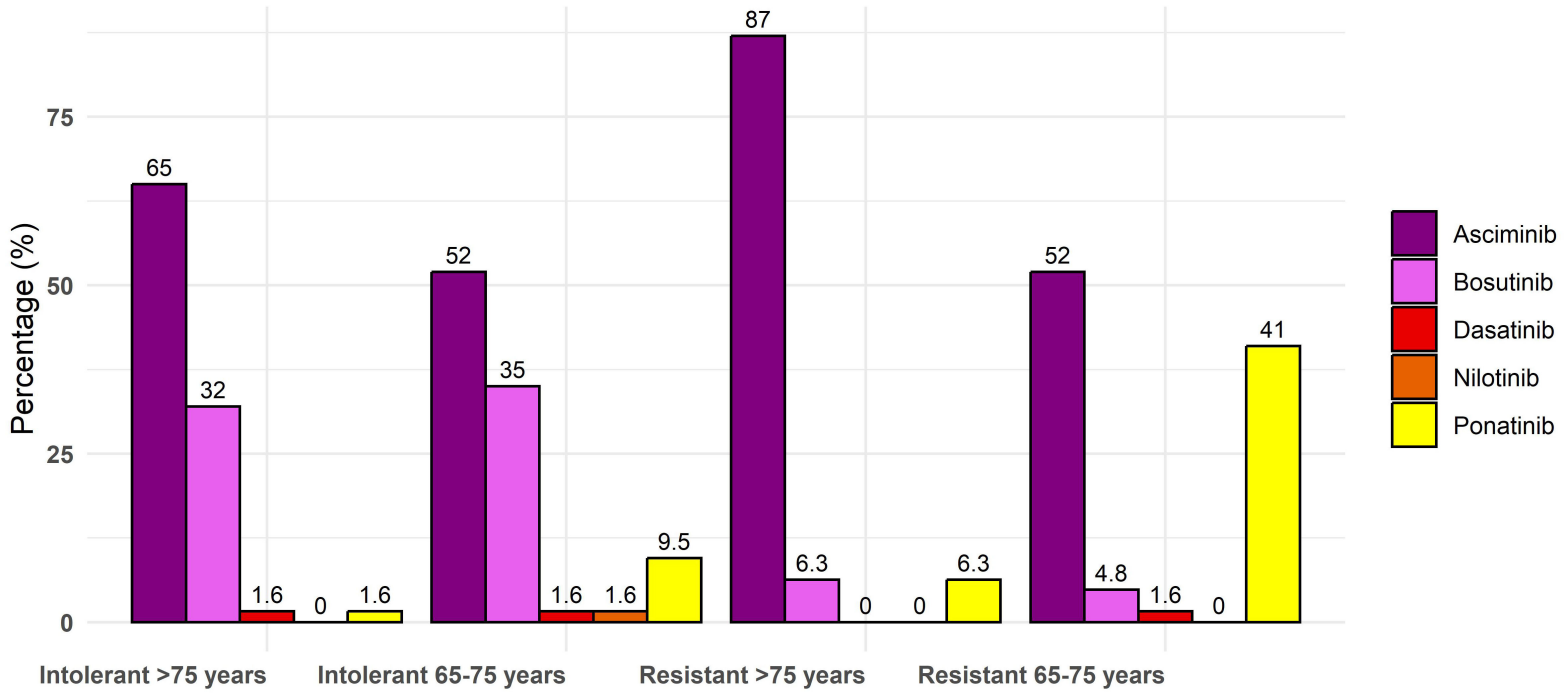
Percentage of each drug in third line for elderly patients for resistance and intolerance.

Considering the possible choice of asciminib as the third-line treatment after failure of imatinib and 2gen TKI, each class of age could benefit from the drug, in particular, very elderly patients aged >75 years (87% of preferred option, n = 55 vs. 6% only of bosutinib and ponatinib each). Indeed, in the subset of resistant patients aged 65–75, asciminib was chosen but without specific differences with ponatinib (52% vs. 41%), with only a few participants indicating dasatinib (1.6%) and bosutinib (4.8%). In truly intolerant patients of each class of age (65–75 or >75 years old), physicians preferred asciminib (**Figure 2**). In intolerant patients aged 65–75 years, asciminib could be preferred by 52% of participants compared to 35% for bosutinib, 9.5% for ponatinib, and 1.6% for nilotinib and dasatinib. In patients aged >75 years, asciminib was preferred by 65% of participants compared to 32% for bosutinib, 1.6% for ponatinib, and dasatinib. Asciminib was preferred also in patients with pre-existing cardiovascular comorbidities by 47 (75%) participants. In this setting, only six physicians (13%) preferred to use the drug at a reduced dose instead of 40 mg twice a day. More than 70% of physicians thought that asciminib could also be an option as a first-line treatment, and they were asked in which subset of patients: 17 (39%) believed that all patients could benefit regardless of the baseline profile and desired final endpoint, 12 (27%) believed that older patients are unfit for other treatments with 2gen TKIs, 10 (23%) believed that younger patients have a future treatment-free remission (TFR), and five (11%) believed that patients regardless of age should not be candidates for discontinuation. The main reason to prefer asciminib as a frontline treatment is due to its tolerability according to 34 (54%) physicians, its efficacy according to 13 (21%) physicians, possible faster responses according to five (7.9%) physicians, and the possible increased rate of discontinuation in the future according to 11 (17%) physicians.

## Discussion

More than 1.5 years has passed since the availability of asciminib as a third-line treatment. A previous survey was conducted when the drug was available only in trials or as compassionate use. The main reason to switch to asciminib in the third or later lines was resistance in more than 70% of the cases. In the present results, asciminib has become the second most used drug for resistance and the first in case of switch for intolerance. We re-proposed the same scenarios to investigate the positioning of the drug in the third line, and the same results were reported in patients treated with imatinib as the frontline treatment and 2gen TKIs as the second-line treatment (from 35% of the previous report to 41%) or after failure of 2gen TKIs as the frontline treatment and rescue with ponatinib as the second-line treatment. As a result of consolidated data with ponatinib and bosutinib as third-line treatments and the availability of asciminib, less than 10% of physicians indicated a switch to a third line with rotation of a 2gen TKI, dasatinib or nilotinib, considering the low rate of responses that can be obtained. Asciminib is available as a third-line treatment after the Food and Drug Administration (FDA) and EMA approval based on the results of the randomized, phase III trial ASCEMBL, which tested the drug versus bosutinib as a third-line treatment. The update at 156 weeks showed definitively the advantage of asciminib in terms of molecular responses with manageable toxicity, represented mostly by thrombocytopenia and increased lipase level (5). The efficacy of later lines has been confirmed by real-world data extrapolated by managed access programs (MAPs) of different countries (10–13). In Italy, 77 patients were treated with the drug in this program, with faster responses after 3 months and an advantage for ponatinib-naïve patients (13). Most of the Italian patients enrolled were heavily pre-treated with three or more TKIs, and 49% received ponatinib as the last drug. Of them, 57% were switched to asciminib due to resistance; as best response, 53% of patients achieved at least an MMR and 32.5% a deep molecular response. No differences were revealed in the response rate among resistant and intolerant patients. Five out of 11 patients with the T315I mutation obtained an improvement at an increased dose of 200 mg BID. Reduced responses and different outcomes after a previous treatment with ponatinib have also been reported in other MAP reports (10–12). A direct comparison between the two drugs has never been performed, but two matched analyses were reported. The first matched analysis, including four different sponsored trials, showed that in patients without baseline response of *BCR::ABL1*^IS^ ≤ 1%, the adjusted difference between ponatinib and asciminib was 9.33% by 12 months in favor of ponatinib. In patients with the T315I mutation, the adjusted *BCR::ABL1*^IS^ ≤ 1% rate difference with ponatinib vs. asciminib was 43.54% by 12 months. The results of this matching-adjusted indirect comparison (MAIC) suggest an increased efficacy of ponatinib, with a high response rate in T315I-mutated patients (14). Indeed, another propensity score matching analysis, including only patients treated with ponatinib and asciminib outside clinical trials, reported that 607 patients were collected, and 270 were extrapolated after matching. The primary endpoint of the analysis was the estimation of failure-free survival at 1 year. Patients were matched according to age, history of cardiovascular disease, the disease phase, the presence of a T315I mutation, and the reason for failure to prior TKI. From the analysis, it emerged that asciminib had increased failure-free survival (45.4% vs. 29.8%), with 50% increased risk of failure of ponatinib; a profile of patient candidates to asciminib was also suggested because it seems that the drug offers major advantages in resistant patients with a baseline BCR::ABL1 ratio < 10%, regardless of previous cardiovascular history (15). Asciminib is approved in the USA as a frontline treatment after the results of the sponsored ASC4FIRST trial, which tested the drug vs. all other available selected TKIs. The first follow-up showed a significant difference in MMR rate in favor of asciminib compared to all TKIs and imatinib (8). The FDA approved the drug with the evidence of an advantage regardless of baseline features [age, gender, EUTOS long-term survival (ELTS) risk, and cardiovascular conditions] and a low rate of discontinuation due to adverse events (only 5% compared to approximately 13% with imatinib and second-generation TKIs). In the last follow-up at 96 weeks, the advantages were confirmed for the primary endpoint: compared to all available TKIs, the MMR rate was 74.1% vs. 52% with a difference of 22.4%; compared to imatinib, the MMR rate was 76.2% vs. 47.1%. The prolonged follow-up also observed an initial difference with 2gen TKIs (72% vs. 56.9%). The most relevant side effects reported were thrombocytopenia and increased lipase level (16). The efficacy and tolerability of asciminib as a first line were also confirmed by the new results of the ASC4START trial: 568 newly diagnosed patients were randomized to asciminib 80 mg QD vs. nilotinib with the primary endpoint of time to treatment discontinuation due to adverse events (TTDAE). After a median follow-up of 9.7 months, the molecular responses were in favor of asciminib (MMR 22.9% vs. 10.2%, MR4 4.6% vs. 1.1%, and MR4.5 2.5% vs. 0.4%). The primary endpoint was achieved: fewer discontinuations were observed in the asciminib arm (5.6%) compared to the nilotinib arm (12.1%) (17). From the results of this survey, it seems that Italian physicians are ready to use it in newly diagnosed patients regardless of the initial patient clinical findings, in both younger and elderly patients, and the principal reason for this choice is the tolerability. The results of this survey should be interpreted with the limitations of a study with aggregate data, considering the approval of asciminib as a third-line treatment, but not as a first-line treatment. Probably, the therapeutic algorithms will change in the next few years with all available TKIs that will become generics. This research can help to highlight the possible changes in the current clinical scenario.

In conclusion, the survey showed that the introduction of the new allosteric inhibitor changed the current algorithm of treatment, in either resistant or intolerant later lines of CML. In the near future, this survey will be re-proposed because it represents a valid tool to understand changes in the therapeutic scenario. The reduction of the dose for other available TKIs (ponatinib and bosutinib) has become a current strategy to maintain efficacy while reducing the occurrence of side effects, even in later lines. Asciminib, as a possible frontline treatment, has been well-received by most of the participants, who believed that this new drug could increase the pharmacological options for most patients.

## Data Availability

The raw data supporting the conclusions of this article will be made available by the authors, without undue reservation.
